# Monolithically Integrated Mid-Infrared Quantum Cascade Laser and Detector

**DOI:** 10.3390/sl30202196

**Published:** 2013-02-06

**Authors:** Benedikt Schwarz, Peter Reininger, Hermann Detz, Tobias Zederbauer, Aaron Maxwell Andrews, Werner Schrenk, Gottfried Strasser

**Affiliations:** Institute for Solid State Electronics and Center for Micro- and Nano structures, Vienna University of Technology, 1040 Vienna, Austria; E-Mails: peter.reininger@tuwien.ac.at (P.R.);hermann.detz@tuwien.ac.at (H.D.); tobias.zederbauer@tuwien.ac.at (T.Z.);aaron.andrews@tuwien.ac.at (A.M.A.); werner.schrenk@tuwien.ac.at (W.S.);gottfried.strasser@tuwien.ac.at (G.S.)

**Keywords:** quantum cascade laser, quantum cascade detector, sensing, lab-on-a-chip, mid-infrared spectroscopy, monolithic integrated, bi-functional

## Abstract

We demonstrate the monolithic integration of a mid-infrared laser and detector utilizing a bi-functional quantum cascade active region. When biased, this active region provides optical gain, while it can be used as a detector at zero bias. With our novel approach we can measure the light intensity of the laser on the same chip without the need of external lenses or detectors. Based on a bound-to-continuum design, the bi-functional active region has an inherent broad electro-luminescence spectrum of 200 cm^−1^, which indicate sits use for single mode laser arrays. We have measured a peak signal of 191.5 mV at theon-chip detector, without any amplification. The room-temperature pulsed emission with an averaged power consumption of 4 mW and the high-speed detection makes these devices ideal for low-power sensors. The combination of the on-chip detection functionality, the broad emission spectrum and the low average power consumption indicates the potential of our bi-functional quantum cascade structures to build a mid-infrared lab-on-a-chip based on quantum cascade laser technology.

## 1. Introduction

Mid-infrared (MIR) laser spectroscopy is an extremely useful tool to identify chemical and biological substances. In this so called “fingerprint” region (3–20 μm) most molecules have their vibrational and rotational resonances, which can be observed by narrow optical absorption lines or changes of the refractive index. Much effort has been devoted to reducing the size of spectroscopic setups down to chip-scale dimensions. However, the goal of monolithic integration has not been reached yet. To build monolithic integrated photonic sensors, it is necessary to emit and detect light with the proper wavelength, as well as to provide sufficient interaction with the observed substance. Although all three components are demonstrated on chip-scale, their integration on a single chip is rather a new topic.

Quantum cascade lasers (QCLs) are known as one of the most important and influential laser sources for mid-infrared spectroscopy [1,2]. A QCL is a unipolar semiconductor laser based on intersubband transitions in a periodically repeated quantum well structure. Their demonstrated narrow spectral line width and the possibility of choosing the emission wavelength in a broad spectral range in 2.5–300 μm by quantum engineering are key properties. Mid-infrared QCLs are compact devices that can emit at room-temperature with high power levels [3]. QCLs can achieve a wall-plug efficiency exceeding50 % [4,5] and can be designed to cover a broad spectral range, while emitting spectrally single mode. This is important for chemical sensing to identify different molecules with great selectivity and facilitated calibration. This can be realized by a bound-to-continuum design, which inherently has a broad gain spectrum, leading to an electro-luminescence spectrum as broad as 295 cm^−1^[6]. The gain bandwidth can be further increased with multiple strongly coupled upper laser levels [7] or by using heterogeneously designed cascades [8]. With this approach, a tunable single mode laser emission beyond 400 cm^−1^ was demonstrated using an external cavity [9]. To achieve more compact sensors,distributed feedback ridge arrays or surface emitting ring cavity arrays, with a reported spectral range of 220 cm^−1^ and 180 cm^−1^ in [10] and [11] can be used. DFB arrays are perfect for in-plane on-chipsensing, where the surface emitting rings have the advantage a smaller divergence < 3 ° and therefore do not necessarily require external lenses in miniaturized systems [12]. Alternatively, single mode emission can be achieved without gratings utilizing coupled ridge cavities [13], a coupled active ring resonator [14] or Mach–Zehnder interferometer type cavities [15]. Without the need of high quality lithography, their processing is the same as for a simple ridge laser. These methods are thus promising forcost-effective devices.

Commonly, MIR spectroscopy setups are realized using broadband MCT-detectors (HgCdTe).More recently, spectroscopic setups have been realized using quantum cascade detectors QCDs [16].As indicated by its name, QCDs are the counterpart of QCLs, based on the same principle [17]. Without the need of cryogenic cooling QCDs are ideal for portable low-power applications. Furthermore, the response of QCDs is extremely fast and can be used to observe the peak power of nanosecond laser pulses [18]. This helps to reduce the total power consumption below 10 mW, when the laser operates in pulsed mode. The fact that QCDs can operate at room temperature and are based on the same principle as QCLs makes them a perfect candidate for integration.

As the total absorption scales with the interaction length, it is sometimes difficult to observe measurable signals from light interactions with molecules at chip-scale dimensions. Especially for spectroscopy of chemicals in gas phase or very dilute solutions, an interaction length of several tensor hundreds of micrometer is not sufficient. Therefore much effort has been put into developing advanced on-chip sensing concepts, to enhance the light matter interaction for possible on-chip sensing applications [19]. This can be realized by using cavity-enhanced absorption spectroscopy [20],photonic crystal cavities [21], intra-cavity absorption spectroscopy [22], by detecting a refractive index change [23,24] or by using plasmonic resonances [25]. For on-chip sensing of fluids, things become much easier, since liquid phase absorption is much higher than in gas phase, while at the same time the absorption features are broadened and therefore do not require a high spectral resolution. Furthermore,the microfluidics are a well developed technology that can be used e.g., in combination with DF Bridge arrays, as reported in [23]. If the fluid is put in the vicinity of the laser, the effective index of the fluid influences the Bragg wavelength of the DFB grating and thus the emission wavelength. An additional sensing method is based on chemically sensitive layers that change their optical properties if the environment is changed. This can be exploited by using a pH sensitive layer, which induces an emission wavelength shift of a QCL [26]. However, all of these chip-scale sensing concepts were demonstrated with external optics and detectors.

In this paper we demonstrate the monolithic integration of a QCL and a QCD. The device is based on our recently developed bi-functional quantum cascade laser and detector (QCLD) active region [27].As our approach is compatible with QCL chip-scale sensing concepts, we move a significant step towards a mid-infrared lab-on-a-chip. After a brief review of the principal function of QCLs and QCDs as well as the main design issues of a bi-function active region in Section 2, we follow with the emission and detection of mid-infrared light on the same chip in Section 3.

## 2. Bi-Functional Quantum Cascade Devices

In this section, we introduce concept of our bi-functional quantum cascade active region, which is a laser when biased and a detector at zero bias.

**Figure 1.**
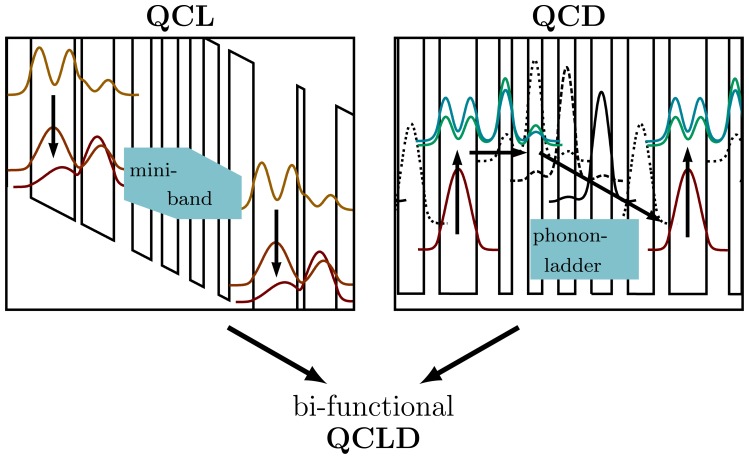
Schematic conduction band structure of a QCL and a QCD. The functionality of these devices is combined into our QCLD, a bi-functional active region for same-frequency lasing and detecting.

Figure 1 illustrates the principle of a QCL and a QCD. In a QCL, the conduction band profile appear stilted due to the applied bias and therefore enables electrons to be injected from a mini-band into the upper laser level by tunneling. These electrons are then relaxed both radiatively and non-radiatively(which is undesirable) to the lower laser level. For optical gain it is necessary to provide population inversion, meaning that more electrons are in the upper than in the lower laser level (neglecting k-space).This is commonly realized by fast extraction of electrons from the lower laser level via multiple extraction levels to the next mini-band utilizing resonant LO-phonon scattering. This process is repeated once per period, where the total number of periods in this work was chosen to be 35. As the band-diagramis determined by the thickness and height of the individual quantum well and barrier layers, QCLs are extremely flexible devices that can be engineered in a wide range in terms of wavelength, optical power,operating temperature, wall-plug efficiency, wavelength tuning range and many more.

QCDs commonly operate at zero bias or a small reverse bias [28]. Near thermal equilibrium most electrons are in the lowest energy level, which is the lower detector state. When illuminating the device at the proper wavelength, electrons are excited to the upper detector level by absorbing photons. With a finite probability these electrons are extracted via tunneling followed by LO-phonon scattering via the phonon-ladder down to the lower detector level of the next cascade. This process is repeated for each of the 35 cascades. In this case one electron has been moved across the structure requiring at least 35 photons that are absorbed, which reduces the responsivity. However, the benefit over common photoconductive QWIPs lies not in the high responsivity, but in the superior noise behavior of the QCDsat higher temperatures [28]. That makes QCDs an interesting alternative for sensing applications. In contrast to common photoconductive QWIPs, QCDs can operate at zero bias in the Johnson noise limit with negligible dark-current noise and without capacitance saturation in the readout circuit [17]. The small dimension, the design flexibility as well as the fact that QCDs are based on the same technology as QCLs are important issues for integration.

In principle, a common QCL can be also used as a detector, but it would detect at a wavelength different from the lasing wavelength with a poor performance. Hofstetter *et al.*[29] have reported a responsivity of 50 μA/W and 120 μA/W using a 9.3 μm bound-to-continuum and a 5.3 μm two-phononresonance QCL structures as photodetectors. In our recent publication [27], we have shown that it is possible combine the functionality of a QCL and a QCD within the same active region. We have matched the emission and detection wavelength, while providing a desirable room-temperature performance for both the laser and the detection functionality. We have reported a room temperature detection from 5.7to 7.2 μm with a peak responsivity of 3.6 mA/W, which is a good value for a pure QCD [17]. At laser operation, the device emits multi mode in the range of 6.4 to 6.8 μm at a power of 45 mW with 100 nspulses at 5 kHz. The active region has been designed that the detector spectrum covers the laser spectrum at room temperature. This has been achieved by reducing the coupling between the lower laser level and the extraction levels with thicker barriers and by inserting a narrow well between the active well and the injector barrier. With the precise adjustment of this narrow well, it is possible to match the emission and detection wavelength at a specified operating temperature. In a second step an injector has been designed that acts as a phonon-ladder based extractor at zero bias. As this design process is rather complicated,we have developed an optimization tool based on a semi-classical Monte-Carlo transport simulator [30].The design approach results in a bound-to-continuum design that intrinsically provides a broad gain,which is desirable for spectroscopic applications to cover a wide range of absorption lines. As discussed in the introduction, individual lines can be addressed by the use DFB gratings, coupled cavities, coupled ring resonators and some more.

## 3. On-Chip Lasing and Detection

Bi-functional quantum cascade laser and detector structures open new possibilities for compact miniaturized mobile mid-infrared sensors. In this section, we present the monolithic integration of aquantum cascade laser and detector, based on our bi-functional active region.

**Figure 2.**
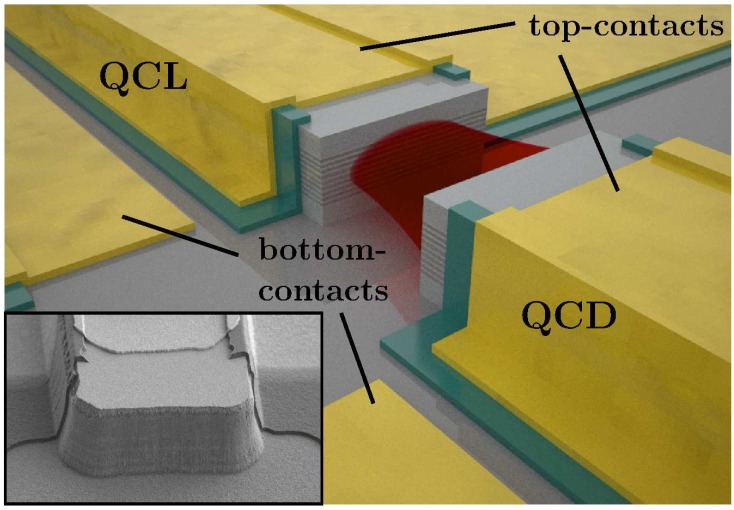
Sketch of the monolithic integrated QCL and QCD. The inset shows a SEM image of the etched laser facet of the 15 μm wide ridge. The gap between the laser and the detector is 10 μm.

The quantum cascade structure was grown by MBE from a slightly strain compensated In GaAs/InAlAs heterostructure on a n-doped InP substrate, with a plasmon-enhanced InGaAs/InAlAs waveguide. The 15 μm wide ridges as well as the 10 μm gap between the laser and the detector were etched by reactive ion etching with SiCl_4_/Ar using a SiN hardmask. We have etched 5.8 μm deep through the active region down to the substrate. The ridges were then passivated with 300 nm SiN, except the contact regions and the facets. We have used extended contacts on the topside for all contacts, instead of a top-contact and a large area contact on the bottom-side of the substrate. Ge/Au/Ni/Au ohmic contacts were evaporated on the ridge and in the adjacent trench for the contacts, to minimize the voltage drop of the detected signal at the semiconductor-metal interface. Extended Ti/Au contacts were then sputtered to provide adhesion on the SiN passivation layer and a better coverage of the sidewalls due to resputtering.Figure 2 shows a sketch of the fabricated device and a SEM image of the etched laser facet. The laser ridge has been cleaved at the other side to 2 mm. The detector ridge is 0.5 mm long with etched facets on both sides.

**Figure 3.**
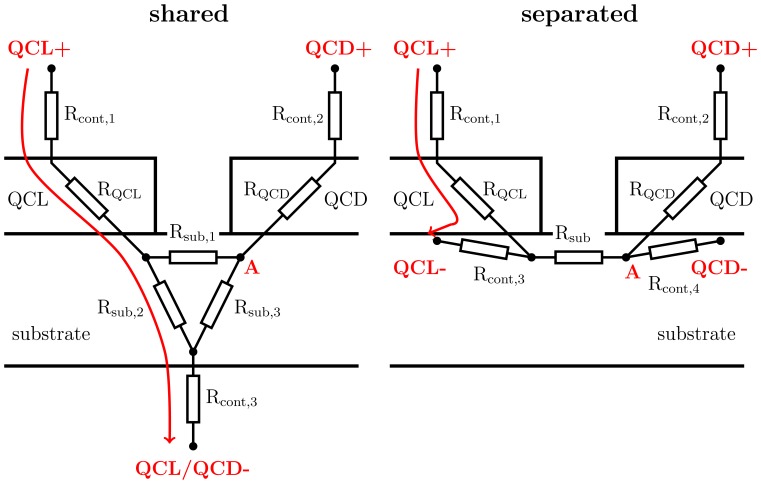
Schematic DC circuit diagram of the device with a shared bottom-side contact **(left)** and separated bottom contacts **(right).** The laser bias voltage is indicated by a red arrow. Separated bottom contacts allow to minimize electrical crosstalk to the detector, as both detector contacts are affected in the same manner. Thus a potential fluctuation at node“A” cancel out.

When using a large area contact on the bottom-side of the substrate, both the laser and the detector would be connected via this contact, leading to electrical crosstalk. As depicted in Figure 3(left), an applied voltage between the laser top contact and the shared bottom-side contact would result in a change of the electric potential at node “A” and thus of the potential at the detectors top contact. Even a small voltage drop at the substrate and the shared bottom-contact would have a significant impact as the detector signal is a factor of hundred smaller than the laser bias. To minimize this effect, we have reduced the shared series resistance by separating the bottom-contact of the laser and the detector. As illustrated in Figure 3(right), an applied voltage again affects the potential at node “A”. Different to before, a potential fluctuation at node “A” changes the potential of both QCD contacts in the same way and thus cancels out.

**Figure 4.**
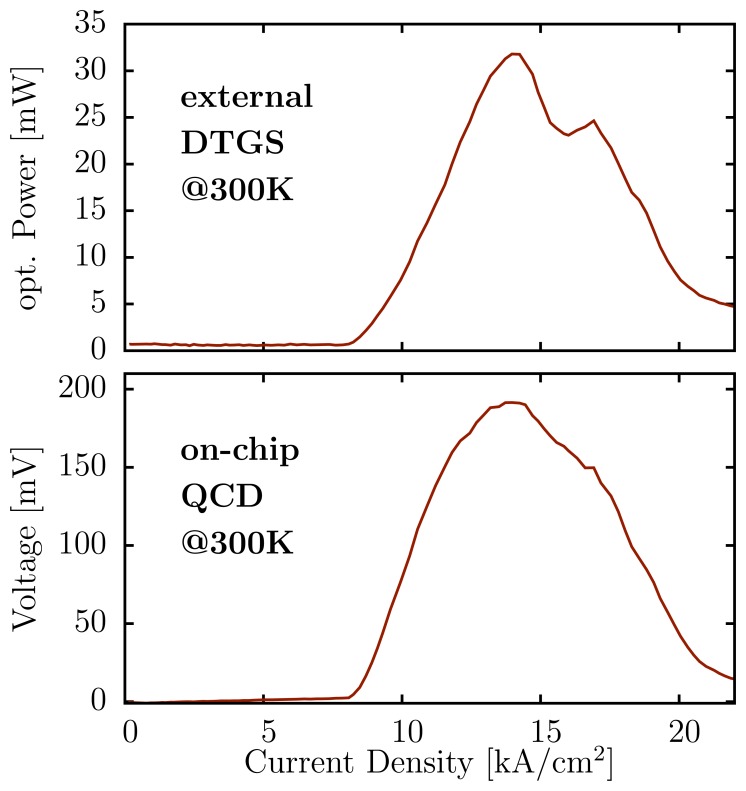
Optical power versus current density of the laser measured with a calibrated external triglycine sulfate pyroelectric detector (DTGS) at the front-facet and the on-chipQCD at the back-facet. All components were operating at room temperature in atmosphere. The laser was operated in pulsed mode with 40 ns pulses at 5 kHz.

Figure 4 shows the light power versus current density plot of the laser, comparing an external triglycine sulfate pyroelectric detector (DTGS) and the on-chip detector. We have observed a detector signal of 191.5 mV at a pulsed peak power of around 30 mW for the on-chip QCD located at the other facet of the laser ridge. The signal was measured with a standard digital oscilloscope without any additional amplifier. This reduces the need of complex measurement circuits, which allows to reduce the size and costs. Due to the fast response of the QCD, the laser pulse duration can be reduced to 40 ns without decreasing the peak detector signal. At a laser bias of around 10 V our device consumes an average power of around 4mW, which is ideal for portable sensors.

**Figure 5.**
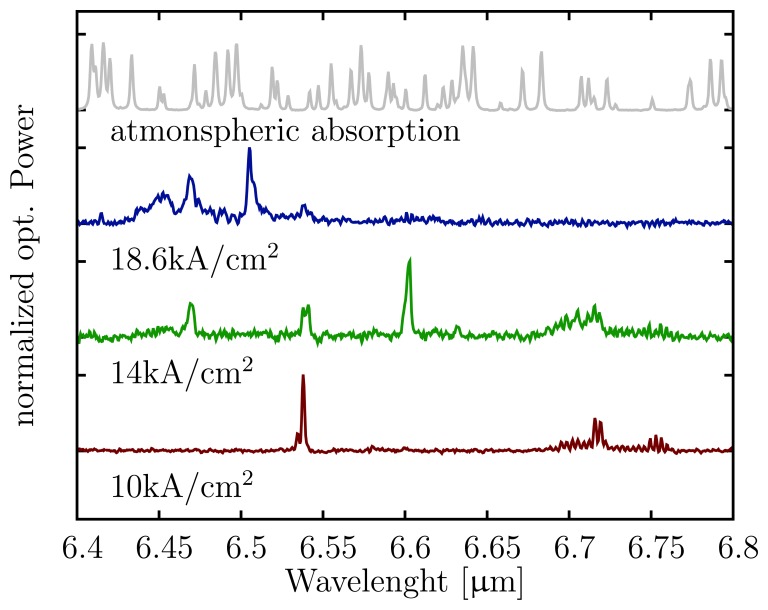
Atmospheric absorption and emission spectra of the QCL at different current densities.

With the separation of the bottom contacts, we were able to reduce the electric cross-talk from the laser to the detector below 2 mV at laser threshold. The remaining cross-talk is mainly due to the external measurement setup and can be eliminated by using a differential measurement approach or by signal post processing, as the crosstalk does not change from pulse to pulse. The DTGS signal was measured with lock-in technique collecting the light of the front-facet of the same laser ridge. In contrast to the lock-intechnique and the slow response of the DTGS, where the average pulse power is observed, the pulsepeak power was measured with the fast QCD. This and the different reflectivity of the two laser facets result in different shapes for both curves depicted in Figure 4. The drop between 14 to 17 kA/cm^2^ is mainly due to atmospheric absorption within the optical path between the laser, the lenses and the DTGS detector. The emission spectra of the QCL at different current densities are plotted in Figure 5. The emission wavelength shifts to longer wavelengths with increasing bias and passes several atmospheric absorption lines. Additionally, the wavelength dependent responsivity of the QCD or the change of the far-field due to excitation of different modes can affect the shape. As our bi-functional device is based on a bound-to-continuum transition, it has an inherent broad emission of 80 cm^−1^, with a Fabry–Pérotcavity. As reported in [27] we have observed a 200 cm^−1^ broad electro-luminescence spectrum with the same active region. This indicates the potential of our device to build a single mode laser array covering a broad spectral range.

## 4. Conclusions

Much effort has been devoted to develop compact miniaturized mid-infrared sensors for mobile applications. However, previously demonstrated QCL based mid-infrared sensing concepts were all realized with external detectors. With the same-frequency emission and detection of mid-infrared light,utilizing the same epitaxial material, our bi-functional structure is a perfect technology platform formid-infrared on-chip spectroscopy. Based on this bi-functional active region, we have fabricated amonolithic integrated laser and detector. We have measured an on-chip detector signal of 191.5 mV at room temperature without any amplification. Since crosstalk is a main issue, it is important to decouple the detector from the laser electrically. This has been achieved by separating both contacts of the lase rand the detector. The electric crosstalk has been reduced below 2 mV and can be further decreased by a differential measurement approach or by signal post-processing. As our approach is compatible with already established on-chip sensing concepts, we move a significant step towards monolithic integrated photonics. The structure can be used as a mid-infrared sensor when combined with microfluidic channels or gas flow cells. Due to the broad optical gain, our active region can be combined with single mode cavity arrays to give spectral resolved information of different chemical substances with great selectivity. Alternatively, it can be used for on-chip power monitoring of QCLs.

## References

[1] Kosterev A.A., Tittel F.K. (2002). Chemical sensors based on quantum cascade lasers. IEEE J. Quant. Electron..

[2] Curl R.F., Capasso F., Gmachl C., Kosterev A.A., McManus B., Lewicki R., Pusharsky M., Wysocki G., Tittel F.K. (2010). Quantum cascade lasers in chemical physics. Chem. Phys. Lett..

[3] Bai Y., Slivken S., Darvish S.R., Haddadi A., Gokden B., Razeghi M. (2009). High power broad area quantum cascade lasers. Appl. Phys. Lett..

[4] Liu P.Q., Hoffman A.J., Escarra M.D., Franz K.J., Khurgin J.B., Dikmelik Y., Wang X., Fan J.-Y., Gmachl C.F. (2010). Highly power-efficient quantum cascade lasers. Nat. Photon..

[5] Bai Y., Slivken S., Kuboya S., Darvish S.R., Razeghi M. (2010). Quantum cascade lasers that emitmore light than heat. Nat. Photon..

[6] Wittmann A., Gresch T., Gini E., Hvozdara L., Hoyler N., Giovannini M., Faist J. (2008). High-performance bound-to-continuum quantum-cascade laser for broad-gain applications. IEEEJ. Quant. Electron..

[7] Yao Y., Wang W., Fan J.-Y., Gmachl C.F. (2010). High performance “continuum-to-continuum”quantum cascade lasers with a broad gain bandwidth of over 400cm^−1^. Appl. Phys. Lett..

[8] Gmachl C., Siva D.L., Colombian R., Capasso F., Co A.Y. (2002). Ultra-broadband semiconductor laser. Nature.

[9] Hugi A., Terrazzo R., Bonnet Y., Wittmann A., Fischer M., Beck M., Faist J., Gini E. (2009). External cavity quantum cascade laser tunable from 7.6 to 11.4μm. Appl. Phys. Lett..

[10] Lee B.G., Hang H.A., Palülg C., Diethyl L., Bellini M.A., Fischer M., Wittmann A., Faist J., Capasso F. (2009). Broadband distributed-feedback quantum cascade laser array operating from8.0 to 9.8μm. IEEE Photon. Technology. Lett..

[11] Mutagenć E., Schwarzenegger C., Yao Y., Chen J., Gmachl C., Strasbourg G. (2011). Two-dimensional broadband distributed-feedback quantum cascade laser arrays. Appl. Phys. Lett..

[12] Mujagić E., Hoffman L.K., Schaeffer S., Mobile M., Schroeder W., Semtex M.P., Wieland M., Massenet W.T., Strasbourg G. (2008). Low divergence single-mode surface emitting quantum cascade ring lasers laser arrays. Appl. Phys. Lett..

[13] Hvozdara L., Lug stein A., Giantkiller S., Schroeder W., Strasbourg G., Entertainer K., Bernoulli E., Goring E. (2000). Self-aligned coupled cavity GaAs/Alga As midinfrared quantum-cascade laser. Appl. Phys. Lett..

[14] Semmel J., Kaiser W., Hofmann H., Höfling S., Forchel A. (2008). Single mode emitting ridge waveguide quantum cascade lasers coupled to an active ring resonator filter. Appl. Phys. Lett..

[15] Liu P.Q., Wang X., Gmachl C.F. (2012). Single-mode quantum cascade lasers employing asymmetric Mach-Zehnder interferometer type cavities. Appl. Phys. Lett..

[16] Hofstetter D., Francesco J.D., Hovzdara L., Herzig H.-P., Beck M. (2011). CO_2_ isotope sensor using a broadband infrared source, a spectrally narrow 4.4μm quantum cascade detector and a fourier spectrometer. Appl. Phys. B..

[17] Giorgetta F.R., Trumann E., Graff M., Yang Q., Manz C., Kohler K., Beere H.E., Ritchie D.A., Linfield E., Davies A.G. (2009). Quantum cascade detectors. IEEE J. Quant.Electron..

[18] Hofstetter D., Graf M., Aellen T., Faist J., Hvozdara L., Blaser S. (2006). 23 GHz operation of a room temperature photovoltaic quantum cascade detector at 5.35 μm. Appl. Phys. Lett..

[19] Monat C., Domachuk P., Eggleton BJ (2007). Integrated optofluidics: A new river of light. Nat. Photon..

[20] Nitowski A., Chen L., Lipson M. (2008). Cavity-enhanced on-chip absorption spectroscopy using microring resonators. Opt. Express.

[21] Mortensen N.A., Xiao S., Pedersen J. (2008). Liquid-infiltrated photonic crystals: Enhanced light-matter interactions for lab-on-a-chip applications. Microfluid. Nanofluid.

[22] Belkin M.A., Loncar M., Lee B.G., Pflügl C., Audet R., Diehl L., Capasso F., Bour D., Corzine S., Hofler G. (2007). Intra-cavity absorption spectroscopy with narrow-ridgemicrofluidic quantum cascade lasers. Opt. Express.

[23] Diehl L., Lee B.G., Behroozi P., Loncar M., Belkin M.A., Capasso F., Aellen T., Hofstetter D., Beck M., Faist J. (2006). Microfluidic tuning of distributed feedback quantum cascade lasers. Opt. Express.

[24] Loncar M., Lee B.G., Diehl L., Belkin M., Capasso F., Giovannini M., Faist J., Gini E. (2007). Design and fabrication of photonic crystal quantum cascade lasers for optofluidics. Opt. Express.

[25] Dey D., Kohoutek J., Gelfand R.M., Bonakdar A., Mohseni H. (2010). Integration of plasmonicantenna on quantum cascade laser facets for chip-scale molecular sensing. IEEE Sens..

[26] Basnar B., Schartner S., Austerer M., Andrews A.M., Roch T., Schrenk W., Strasser G. (2008). Reversible switching of quantum cascade laser-modes using a pH-responsive polymeric claddingas transducer. Opt. Express.

[27] Schwarz B., Reininger P., Detz H., Zederbauer T., Andrews A.M., Kalchmair S., Schrenk W., Baumgartner O., Kosina H., Strasser G. (2012). A bi-functional quantum cascade device for same-frequency lasing and detection. Appl. Phys. Lett..

[28] Gomez A., Carras M., Nedelcu A., Costard E., Marcadet X., Berger V. (2008). Advantages of quantum cascade detectors. Proc. SPIE.

[29] Hofstetter D., Beck M., Faist J. (2002). Quantum-cascade-laser structures as photodetectors. Appl. Phys.Lett..

[30] Baumgartner O., Stanojevic Z., Kosina H., Sabelfeld K.K., Dimov I. (2012). Monte Carlo Simulation of Electron Transport in Quantum Cascade Lasers. Monte Carlo Methods and Applications.

